# Linear unmixing protocol for hyperspectral image fusion analysis applied to a case study of vegetal tissues

**DOI:** 10.1038/s41598-021-98000-0

**Published:** 2021-09-20

**Authors:** Adrián Gómez-Sánchez, Mónica Marro, Maria Marsal, Sara Zacchetti, Rodrigo Rocha de Oliveira, Pablo Loza-Alvarez, Anna de Juan

**Affiliations:** 1 Department of Chemical Engineering and Analytical Chemistry, Universitat de Barcelona, 08028 Barcelona, Spain; 2 ICFO- Institut de Ciències Fotòniques, The Barcelona Institute of Science and Technology, 08860 Castelldefels, Spain; 3 Department of Chemistry, Università Di Roma “La Sapienza”, 00185 Rome, Italy

**Keywords:** Analytical chemistry, Fluorescence spectroscopy, Infrared spectroscopy, Raman spectroscopy, Imaging and sensing, Scientific data

## Abstract

Hyperspectral imaging (HSI) is a useful non-invasive technique that offers spatial and chemical information of samples. Often, different HSI techniques are used to obtain complementary information from the sample by combining different image modalities (Image Fusion). However, issues related to the different spatial resolution, sample orientation or area scanned among platforms need to be properly addressed. Unmixing methods are helpful to analyze and interpret the information of HSI related to each of the components contributing to the signal. Among those, Multivariate Curve Resolution-Alternating Least Squares (MCR-ALS) offers very suitable features for image fusion, since it can easily cope with multiset structures formed by blocks of images coming from different samples and platforms and allows the use of optional and diverse constraints to adapt to the specific features of each HSI employed. In this work, a case study based on the investigation of cross-sections from rice leaves by Raman, synchrotron infrared and fluorescence imaging techniques is presented. HSI of these three different techniques are fused for the first time in a single data structure and analyzed by MCR-ALS. This example is challenging in nature and is particularly suitable to describe clearly the necessary steps required to perform unmixing in an image fusion context. Although this protocol is presented and applied to a study of vegetal tissues, it can be generally used in many other samples and combinations of imaging platforms.

## Introduction

Hyperspectral imaging (HSI) is a useful non-invasive analytical technique that allows preserving the morphological and chemical information associated with samples. This technique consists of collecting spectroscopic information associated with different points (pixels) of a scanned area in a sample. In this way, spatial and chemical information about the samples is provided and limitations linked to traditional single point spectroscopic techniques, such as the lack of spatial information, are clearly overcome. Nowadays, imaging platforms offer a wealth of spatial resolution scales and are adapted to the specificities of many spectroscopic (and spectrometric) modalities^1,2^. Despite the clear value of the complementary information provided by the currently available imaging platforms, image fusion is still a challenge that does not have a generalized solution^3^.

The size and complexity of the information provided by hyperspectral images need powerful chemometric techniques for their adequate interpretation. Very often, the goal of HSI is providing information about the nature and location of sample constituents. In the beginning of hyperspectral imaging, the compound location was described displaying maps at selected spectral channels and the compound spectral fingerprint was associated with spectra of pixels located in specific sample regions. However, such an approach is clearly insufficient for complex multicomponent samples, where often no selective spectral channels exist and the extraction of clear compound fingerprints is hindered by the colocation of components in the pixels of the image. Unmixing methods come then into play to provide pure spectral signatures and pure concentration maps of the image constituents and, hence, a global chemical, quantitative and morphological information of the samples studied.

The unmixing task can be tackled by linear and non-linear methods depending on the underlying model assumed to define the spectroscopic measurement, i.e., the spectroscopic signal in every pixel is defined as a concentration-weighted linear combination of the pure spectra of the image constituents in linear models or the signal definition obeys more complex models in non-linear approximations. Linear unmixing methods reflect exactly the basic form of the spectroscopic Beer-Lambert law, where every component is defined by an invariant spectral fingerprint that contributes to the signal measured proportionally to its concentration. Non-linear methods, instead, take into consideration that there may be variability in the spectral fingerprint of a particular component in certain instances. The non-linear unmixing problem is often solved by using deep learning methods based on the use of neural network autoencoders^4,5^. Such an approach has found applicability basically in remote sensing scenarios, where the definition of component, e.g., soil, vegetation… and the conditions of the image acquisition may sometimes justify the assumption of a certain variability in the spectral signatures of components. However, in a very large number of cases, particularly when image platforms located in the laboratory are used, the results provided by linear unmixing methods are a very good approximation of the real behavior of the spectroscopic measurement, need a lower computation time and allow a simpler implementation of external available information under a variety of constraints^6^. As in any other data analysis context, the parsimony principle stating that the simplest model that provides a satisfactory description of the phenomenon studied has to be chosen prevails in this case and the protocol proposed in this work is based on linear unmixing methodologies.

Within the family of linear unmixing methods, Multivariate Curve Resolution-Alternating Least Squares (MCR-ALS)^7^ is a chemometric method that has been widely used for image analysis due to the flexible characteristics offered in terms of the data structures that can be potentially studied and the diversity of information that the algorithm incorporates under the form of constraints to help in the modelling of concentration maps and spectral signatures of the pure components^7–9^. Other linear unmixing methods, often used in the remote sensing area, tend to work forcing necessarily non-negativity and normalization constraints or using libraries of previously known spectral signatures and do not change the modus operandi when dealing with an individual image or with an image fusion scenario^10^. Hence, the choice of the MCR-ALS method in this work.

MCR-ALS has been successfully applied to the analysis of single images or sets of related images acquired with the same spectroscopic platform^9^. However, the use of this methodology for fusion of images from different platforms is not extended yet, although few examples are reported to address problems such as the fusion of images with different spatial resolutions^11,12^, with spectroscopic modalities showing different dimensions^13^ or with the combination of spectroscopic and color information^12,14^.

In a general multiplatform fusion context, images from each platform could be analyzed separately by MCR-ALS, but the fusion of the information from the different image techniques provides a complete description of the sample constituents and more accurate solutions^3,9^. Multiplatform image fusion allows exploiting the complementary information provided by different spectroscopic techniques and obtaining simpler models including all the gathered information in a single complete, reliable, and robust model to answer to the scientific question of interest. Image fusion has started to emerge as an excellent methodology to analyze data of different chemical systems.

An area where image fusion can be particularly relevant is the research of the structure and composition of tissues in living organisms. Indeed, the natural complexity of the biological tissues looks as a problem that can be adequately addressed with the use of imaging platforms sensitive to different information and components. The results obtained can hopefully provide the necessary link between chemical structure and function required for the understanding of these systems.

An interesting case study of biological interest to apply image fusion strategies is the characterization of vegetal tissues. Indeed, different hyperspectral imaging techniques can be used simultaneously to obtain complementary information for this particular biological system. In this respect, the combination of fluorescence, Raman and infrared imaging techniques is a good option. Fluorescence images collect the emission fluorescence spectra from natural fluorophores in plants, such as lignin, chlorophylls^15^, while Raman images provide information about cellulose, lignin, carotenes and other components^16^. Finally, infrared images provide information about molecular components, such as proteins, lipids and carbohydrates^17^. However, spatial resolution from conventional infrared HSI is insufficient for a good definition of micron vegetal tissue substructures. Synchrotron Radiation Fourier Transform Infrared (SR-FTIR) imaging, instead, has the necessary spatial resolution and helps to reveal the microstructures at tissue level^18^.

In this work, SR-FTIR, Raman and fluorescence HSI from rice leaf cross-sections were acquired to study thoroughly the different constituents and structures found in the tissues of this plant. For the first time, SR-FTIR, Raman and fluorescence HSI are fused and analysed by MCR-ALS. To do so, images from the different platforms had to be balanced in terms of spatial resolution, orientation and area scanned before being analysed. As a result, spectral signatures of plant components showing the relevant features of all different spectroscopic techniques used and the related distribution maps defining accurately the spatial structure of the biological elements identified were obtained. The steps followed and the gain obtained when using image fusion as compared with the analysis of images coming from individual platforms is clearly proven. Despite the intrinsic interest of the characterisation of components in vegetal tissues, the main goal of the work is providing a general framework that can be generally adopted to address linear unmixing in any multiplatform image fusion problem.

## Experimental

### Plant growth and sample preparation

Rice plants were obtained from *Oryza Sativa Japonica Nipponbare* seeds provided by the Center for Research in Agricultural Genomics (CRAG) at Autonomous University of Barcelona. This public university center complies with all necessary legislative regulations on the treatment of plant seeds and living organisms and the seeds used do not present any kind of hazardous risk for their growth and use. Seeds were germinated for two days at 30 °C in a wet environment. After germination, seeds were planted in small individual pots with a universal substrate BATLLE, composed by coconut fiber, peat moss, composted vegetal material and perlite with pH 7.25. Rice plants were watered two times per week with 400 mL of Milli-Q water for 33 days under controlled conditions of temperature, light and humidity in an Environmental Test Chamber MLR-352H (PANASONIC) in the Institute of Environmental Assessment and Water Research-Spanish National Research Council (IDAEA-CSIC).

**Figure 1:**
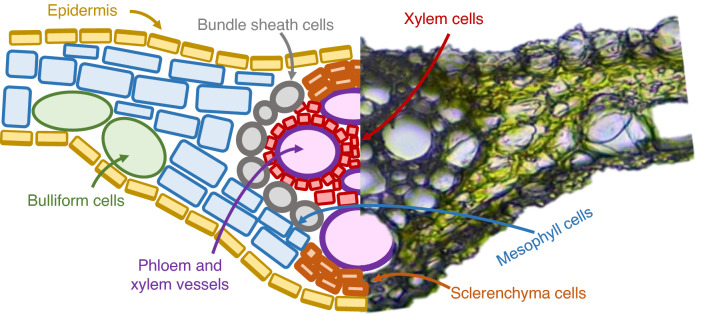
Schematic and optic cross-section image of a rice leaf.

Once the plants were grown, small pieces of plant leaves of different plants were collected and embedded in agarose. Straightaway, three cryosections of seven μm-thickness were obtained using a cryostat at the Parc Científic of Barcelona at − 20 ± 5 °C. Sections were placed on a calcium fluoride slide of 1 mm-thickness, covered with a calcium fluoride coverslide of 0.5 mm-thickness and sealed with nail polish. In every cross-section, different regions can be observed (Fig. 1). Mesophyll cells, where photosynthetic activity is located and chlorophyll or carotenoids can be found. The characteristic green color of the plants comes from this type of cells. Also, the epidermis can be observed. The function of the epidermis is to protect the plant tissue from external damages. Several compounds can be found there, such as resins that cover the epidermis to avoid water loss. In the vascular system, two parts are differentiated: xylem and phloem. Xylem is a type of lignified tissue that transports water and minerals and it is formed by a conglomerate of the bigger channels. Phloem is another type of lignified tissue that transports nutrients as sugar or other biomolecules. It is located on top of the xylem in a cross-section view. Finally, sclerenchyma cells can be located on the top and the bottom of the vascular system. Sclerenchyma cells are strongly lignified cells and give hardness to the plant.

## Image acquisition

### Synchrotron infrared image acquisition

All SR-FTIR HSI were collected at the SYNCHROTRON ALBA (Cerdanyola del Vallès, Catalunya, Spain, MIRAS beamline). The Fourier transform infrared spectrometer used was equipped with a TE Cooled DLaTGS Detector Vertex coupled to a HYPERION 3000 Microscope. The detector of the IR microscope was a liquid-nitrogen-cooled 50 μm HgCdTe detector, covering the range of 10000–600 cm^−1^. The microscope was operating using a 36 × objective. IR spectra were acquired in transmission mode by point mapping and every spectrum was associated with a pixel sized 3 × 3 μm^2^. Spectra were collected in the infrared region covering the range of 4000–1000 cm^−1^ with 4 cm^−1^ resolution and 64 accumulations. Background was collected every 25 spectra with 128 accumulations.

### Fluorescence image acquisition

Fluorescence HSI were collected using a LEICA TCS SP8 STED 3X microscope (LEICA MICROSYSTEMS, Mannheim, Germany). A 405 nm laser beam with a power approximately of 160 μW focused through a 10 × objective LEICA HC Pl Apo was used as a light source. A Gated HyD hybrid detector in photon counting mode was used for the spectra collection. Spectra were collected by laser point scanning with an exposure of 0.825 μs/pixel with 70% of total laser power. Every line is formed approximately by 800 pixels and each line was accumulated two times to improve signal to noise ratio. The studied spectral emission range goes from 420 to 750 nm, with a spectral resolution of 5 nm and the pixel size of 0.25 × 0.25 μm^2^.

### Raman image acquisition

Raman HSI were collected using an INVIA RAMAN Microscope spectrometer (RENISHAW, Gloucestershire, UK). A 532 nm laser beam focused through a 20 × objective Leica (NA = 0.4) with a power of 25 mW was used as a light source. Spectra were collected by point mapping with 0.25 s exposure time and 10 % of total laser power per pixel. The studied spectral range goes from 270 to 2015 cm^−1^, with a spectral resolution of 1.55–1.95 cm^−1^ depending on the Raman shift scanned. Pixel size was of 2 × 2 μm^2^. The Raman spectrum is recorded on a deep depletion charge coupled device (CCD) detector (RENISHAW RenCam).

## Data analysis

### HSI preprocessing

In the case of SR-FTIR images, due to the opacity of the sample in several regions, some IR spectra were saturated. These pixels were removed and not used in further analysis. Also, for all samples, infrared spectra were first cropped within 3000–1200 cm^−1^ spectral range. Wavenumbers out of this range were not used because of the signal saturation observed. Strong baseline artifacts were also detected. The second derivative was applied to the infrared spectra to remove offsets and linear baselines and to enhance the separation of overlapping peaks through the Savitzky-Golay algorithm^19^. From the derivative spectra, only spectral regions with useful information were selected for further analysis (3000 to 2800 cm^−1^ and 1800 to 1360 cm^−1^).

In fluorescence images, emission fluorescence per pixel was low due to the high spatial resolution (pixel was sized 0.25 × 0.25 μm^2^) and to the low quantum yield of natural fluorophores for this instrumental set. To improve the spectroscopic signal quality, spectra of adjacent pixels were binned to create a pixel with a single spectrum (binning). The binning chosen was (3 × 3) pixels, which provided a final pixel size of 0.75 × 0.75 μm^2^. Despite the pixel binning, spatial resolution was still high and the spectral quality was improved.

In Raman images, all spectra showed a high fluorescence baseline contribution due to the natural fluorophores in the rice tissue. Fluorescence baseline interferes with the Raman peaks hiding them and hardening the interpretation. Raman spectra were corrected by Asymmetric Least Squares^20^ to remove fluorescence baselines. Also, cosmic peaks were corrected by interpolation of Raman intensities of nearest channels. In addition, the range 1100 to 1800 cm^−1^ was used for the analyses. An example of raw and preprocessed SR-FTIR, fluorescence and Raman spectra can be found in Fig. S1 in the Supporting Information.

### Image linear unmixing: multivariate curve resolution-alternating least squares (MCR-ALS)

**Figure 2:**
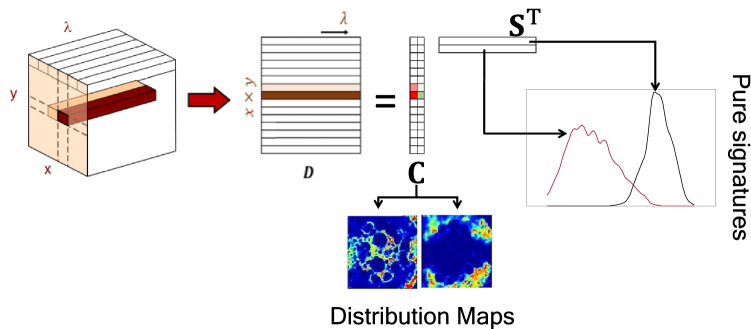
Bilinear model of an HSI.

An HSI can be visualized as a data cube where *x* and *y* are the pixel coordinates and λ the spectral dimension. In this cube, a full spectrum is associated with each pixel coordinate. If the HSI cube is unfolded, the data acquires a matrix structure **D** (*I*×*J*) (Fig. 2) that contains all pixel spectra of the image one under the other. Unmixing methods are able to describe the mixed information in the original pixel spectra in **D** through a bilinear model analogous to the Lambert-Beer law, where the total spectroscopic signal collected can be expressed as the sum of the signal contributions of each individual image constituent. Following the linear Beer-Lambert law, the contribution of each image constituent to the total signal collected can be mathematically expressed by the pure spectrum of the compound **s**_*i*_^T^ weighted by its concentration in the different pixels, **c**_***i***_, defined by the term **c**_***i***_ **s**_***i***_^T^ (Eq. 1). Finally, the typical bilinear model associated with unmixing methods is expressed in compact format as shown in Eq. (2), where the matrix **S**^**T**^ contains the profiles of the pure spectra of the image constituents and the matrix **C** the related concentration profiles. The residuals of the model are expressed by **E** (*I*×*J*). The spectroscopic bilinear model of an image allows expressing the information of every sample constituent with a spectral signature **s**_***i***_^T^ and a concentration profile ***c***_***i***_ that conveniently refolded provides the related distribution map.

1 $${\mathbf{D}} = \mathop \sum \limits_{i} c_{i} s_{i}^{{\text{T}}} + {\mathbf{E}}$$

2 $${\mathbf{D}} = {\mathbf{CS}}^{{\text{T}}} + {\mathbf{E}}$$

Multivariate Curve Resolution-Alternating Least Squares (MCR-ALS) is a multivariate least-squares based iterative resolution (or unmixing) method that alternatingly optimizes matrices **C** and **S**^T^ under the action of constraints that help to provide chemically meaningful spectral and concentration profiles. MCR-ALS is used in many fields of application and is especially suitable for hyperspectral image analysis^7–9^.

The method starts doing an estimation of the number of components present in the original data set **D** by Principal Component Analysis (PCA)^21^ or taking advantage of previous knowledge of the sample (note that when a high number of components is detected in this step, the potential need for a non-linear unmixing method can be considered). Afterwards, an initial estimate of matrix **C** or **S**^T^ (most often spectral estimates in HSI analysis) is built by a pure variable selection method based on Simple-to-use Interactive Self-modelling Mixture Analysis (SIMPLISMA)^22^ or on similar algorithms. Such an estimate and the matrix **D** are used to start the least-squares alternating optimization of the profiles in matrices **C** and **S**^T^ of the bilinear model under the action of constraints until convergence is achieved. The convergence criterion can be a maximum number of iterations or a value related to the difference in fit improvement between consecutive iterations.

The quality of the MCR model fit is described by the lack of fit *LOF* (%), defined by

3 $$LOF\;\left( {\text{\% }} \right) = 100 \times \sqrt {\frac{{\mathop \sum \nolimits_{i,j} e_{ij}^{2} }}{{\mathop \sum \nolimits_{i,j} d_{ij}^{2} }}}$$

and the percent of variance explained, defined by

4 $$r^{2} = { }\left( {1 - \frac{{\mathop \sum \nolimits_{i,j} e_{ij}^{2} }}{{\mathop \sum \nolimits_{i,j} d_{ij}^{2} }}} \right)$$

where *d*_*ij*_ is an element of **D** and *e*_*ij*_ is related to **E**. In a HSI context, when the final bilinear model is obtained, the pure spectral signatures of the image constituents are the profiles in the **S**^T^ matrix and their pure distribution maps can be recovered refolding the related concentration profiles into the original spatial geometry of the image.

**Figure 3:**
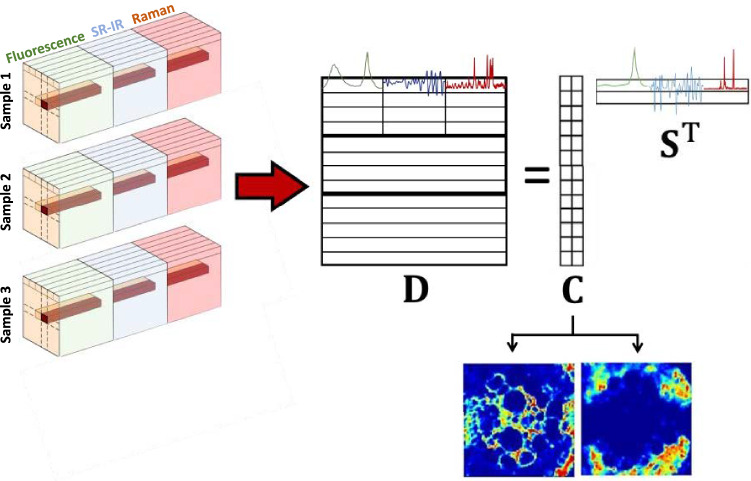
Scheme of the multiset structure issued from image fusion and related bilinear model. In this case, nine data blocks form the multiset: three samples imaged by three spectroscopic platforms.

Often, several images may contain related information. When this is the case, it is possible to build multiset structures that contain several connected images. Multisets can be formed appending blocks of spectra from related images obtained with the same spectroscopic technique on under the other in a column-wise augmented fashion. In this case, the spectral dimension needs to be common for all images. A multiset can also be built appending spectra from images of the same sample obtained with different imaging platforms in a row-wise augmented fashion. In this case, the pixel dimension needs to be common for all images. The most complex and complete multisets can be built connecting images from different samples obtained with different platforms (see Fig. 3) in a row- and column-wise augmented fashion. MCR-ALS can also be used to analyze these multiset structures and a bilinear model is also obtained, where the matrix **C** and/or **S**^T^ can also be formed by small blocks (submatrices) related to concentration profiles of the different images and/or to pure spectral signatures of the different platforms used.

The MCR-ALS analysis of a single image or an image multiset takes the benefit of the use of constraints on **C** or/and **S**^T^ to obtain chemically meaningful and more accurate spectral signatures and distribution maps. Classical constraints, such as non-negativity, are often applied to concentration maps and to some spectroscopic measurements^23^. Another useful constraint is the selectivity/local rank^24,25^. In this context, this constraint may force particular pixels to show null concentration values or some spectral ranges to show null signal for particular image constituents. Such an information can come from previous knowledge or from image-adapted local rank analysis methods^26^. Recently, a new generation of constraints has appeared that takes into account characteristics of the spatial distribution of components as well^27,28^. An asset of the use of constraints in MCR-ALS analysis is that they can be optionally set per component, per mode (**C** or **S**^T^) and per block in each of the **C** or **S**^T^ submatrices in a multiset context. This flexibility allows the preservation of the specific characteristics of the spatial distribution of components in the different samples and the properties of the spectral signatures of the different spectroscopic techniques.

**Figure 4:**
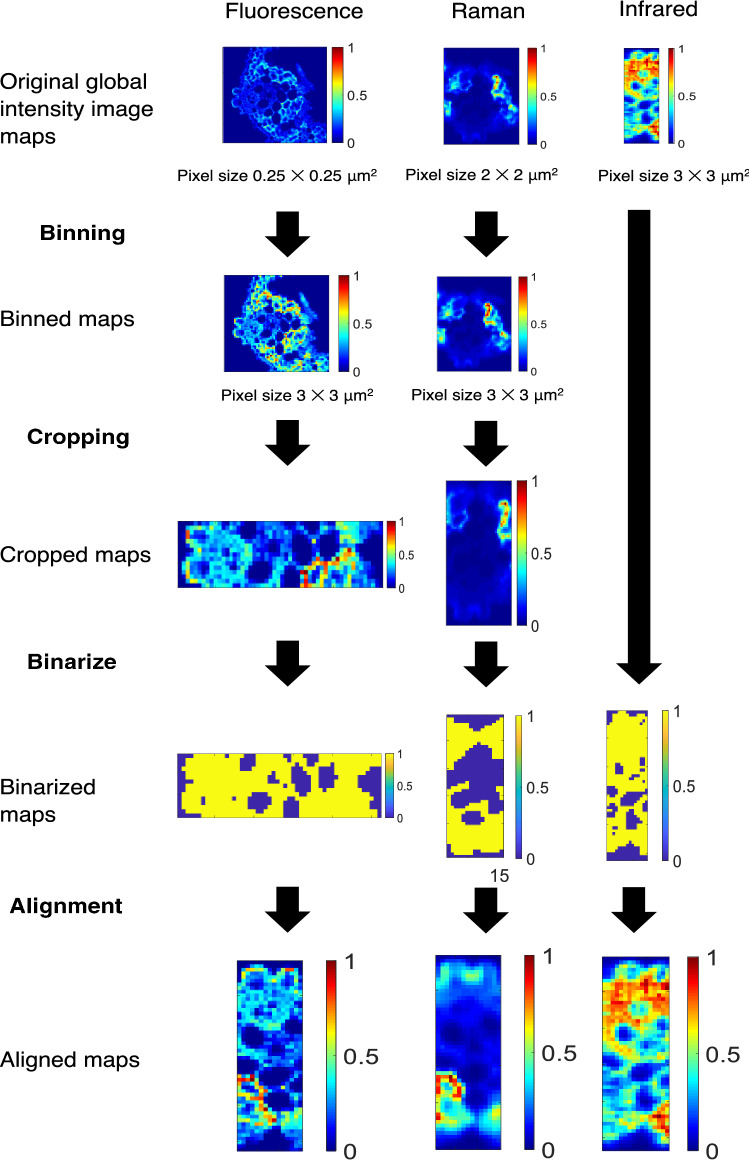
General scheme of the image matching procedure. In order, images are resized until match pixel size. Then, they are cropped until have approximately the same area covered. Next, the images are binarized and aligned among them. Once optimal translational and rotational parameters are achieved, hyperspectral images are aligned.

Studying adequately a multiplatform image multiset passes through the solution of problems linked to the multiset configuration and the multiset analysis. Thus, a proper configuration of the multiset needs a common pixel dimension among the images combined, i.e., having congruent pixels, and an efficient multiset analysis demands a proper balance of the information linked to the data blocks of the different platforms. A proper description of the sequence needed to solve these problems is carried out taking as example the data linked to the case study presented. In this case, the final multiset will have the same structure as shown in Fig. 3 and will be the result of fusing images from three different samples analyzed with Raman, fluorescence and SR-FTIR platforms. This multiplatform image fusion should follow the steps displayed in Fig. 4, which are:

### Building a multiset with congruent pixels

Such a goal requires matching the pixel size of all images and the image area scanned. Afterwards, a spatial transformation (shift and rotation) of images is required to ensure pixel congruency. In the combination of fluorescence, SR-FTIR and Raman images of our case study, this will happen as follows:

#### Matching pixel size of all images and image area scanned

SR-FTIR images are those with largest pixel size, 3 × 3 μm^2^ and smallest area scanned. The rest of imaging techniques are binned to achieve this pixel size. Thus, the pixels of the fluorescence HSI, sized 0.25 × 0.25 μm^2^, were binned by a factor of 12 × 12 to achieve the pixel size 3 × 3 μm^2^. Raman HSI had a pixel size of 2 × 2 μm^2^. An inhouse developed MATLAB script was used to bin and interpolate the pixel values to achieve a 3 × 3 μm^2^-pixel size. Finally, the fluorescence and Raman HSI were also cropped until covering approximately the same area than SR-FTIR HSI.

#### Spatial transformations (shift and rotation of images) for pixel congruency

This step is oriented to compensate the pixel shift and/or rotation among the images to be combined. For this reason, HSI need to be moved in *x* and *y* directions and/or rotated until pixels are congruent among fluorescence, SR-IR and Raman HSI.

To obtain the transforming parameters, binarized maps issued from global intensity maps from each HSI technique can be used. The global intensity maps are 2D representations displaying the sum of all spectral intensities of the channels of each pixel spectrum in the image. Global intensity maps are binarized i.e., pixels are assigned a value equal to one (when signal is significant) or zero (when there is no detectable signal). When images have a clear contour, pixels on the sample have much higher intensity than pixels on the background sample support. This contour shape information can be used for the alignment because all images of the same sample must have the same contour, independently on the spectroscopic techniques used for imaging.

The SR-FTIR binarized map is always taken as reference for the alignment (*Ar*). Sequentially, shifts in in x and *y* and rotation angle θ were computed for fluorescence and Raman images (*As*) with the SR-FTIR reference image. To do that, initial estimates for shifts in *x* and *y* (*dx*, *dy*) and rotation α (*Θ*) are defined and the map of the image to be aligned is modified accordingly. An error function (Eq. 3), defined as:

5 $$ssq\left( {\Theta ,dx,dy} \right) = { }\sum \sum \left( {A_{{i,j{ }\left( {x,y,\alpha } \right)}}^{r} - { }A_{{i,j{ }\left( {x + dx,y + dy,\alpha + \Theta )} \right.{ }\left( {i,j} \right)}}^{s} } \right)^{2}$$

is calculated among the binarized values of common pixels in the image to be aligned and the reference image. This is an iterative process that uses a SIMPLEX optimization algorithm and stops when the error defined in Eq. (3) gets sufficiently small^29^. When shift and rotation parameters are found, the whole HSI is spatially transformed to match the reference image. Only pixels from common sample areas scanned by all techniques are used to create the multiset used for further analysis.

### Balancing the importance of the data blocks related to each platform in the multiset

The pixel spectra provided by the different imaging platforms can show significant differences related to the scale of the signal recorded and to the number of spectral channels in each measurement. If blocks of the raw pixel spectra are appended in the multiset, platforms that provide spectra with higher signal intensity and formed by a large number of spectral channels will have a major influence in the results obtained. A good quantitative representation of the overall signal contribution of an image is provided by the 2-norm of the related unfolded matrix **D**. Hence, to balance the importance of images coming from different platforms on the same sample, the data block of each image will be divided by its 2-norm before the multiset is built. This is a clear mathematical procedure to keep similar the weights of the different blocks of the multiset, less biased than trying to find suitable scaling factors by visual inspection.

Once a balanced and pixel-congruent multiset is built, MCR-ALS can be properly applied setting the appropriate constrains to the profiles in each block of the **C** and/or **S**^T^ matrices. The application of MCR-ALS to analyze single or fused images has been done using a freely downloadable graphical user interface under MATLAB environment that follows the steps described above and provides the possibility to incorporate in a flexible way the suitable constraints^30^.

## Results and discussion

MCR-ALS was used to elucidate the sample constituents present in the cross-sections of rice leaves analyzed by SR-IR, fluorescence and Raman HSI. To show the gain of global information obtained by fusing images from different platforms, two different analyses were performed. On the one hand, three separate multisets (for fluorescence, for SR-IR and for Raman) containing three images each collected with the same platform were structured in a multiset extended in the column-wise direction and were subsequently analyzed by MCR-ALS. This per platform analysis gives a vision of the information that can be obtained without using image fusion. On the other hand, an MCR analysis of a multiset incorporating the images from all platforms (Fig. 3) was carried out to illustrate the gain of information linked to image fusion. To build the fused multiset, the alignment of the images previously described to achieve the congruence of pixels among platforms and the suitable balance between data blocks was carried out.

For all MCR-ALS analyses, the convergence criterion was 0.1% difference among lack of fit between consecutive iterations. The main results of MCR-ALS applied to the different multisets analyzed are summarized in Table 1.

**Table 1:** Summary of MCR-ALS results from the image multisets analyzed.

Multiset	Techniques	Nr. of components	LOF (%)	Explained variance (%)
1	Fluorescence	5	13	98
2	SR-FTIR	3	57	67
3	Raman	4	32	90
4	Fluorescence, SR-FTIR, Raman	6	31	91

As can be seen, the variance explained is satisfactory in all multisets taking into consideration the quality of the spectra analyzed. Thus, SR-FTIR provides the lowest variance explained due to the enhancement of noise when derivative spectra are used. Raman and fluorescence multisets show higher variance explained due to the quality of the original spectra. The analysis of the multiset using all platforms provides a good description of all images analyzed.

As it was expected, the results provided by the imaging platforms used in this work show differences in the number of components modelled due to the complementary information of the related spectroscopic techniques and the differences in the detectable response for the different biological tissues and molecules. These differences suggest the need of a multiplatform fusion to exploit the complementary information and achieve a complete description of the sample.

In the next subsections, a description of the components found by each spectroscopic technique and by the fused multiset containing all platforms is provided.

### Fluorescence HSI multiset analysis

Initial spectral estimates found by a SIMPLISMA-based method pointed out to the presence of components similar to those identified in a previous work^13^. Thus, some components were identified as chlorophylls, which emit in wavelengths higher than 625 nm, lignins in lower wavelengths and an additional component linked to small vesicles, probably oil or silica bodies, inside the mesophyll cells and epidermis in leaves, emitting between these two families of compounds.

**Figure 5:**
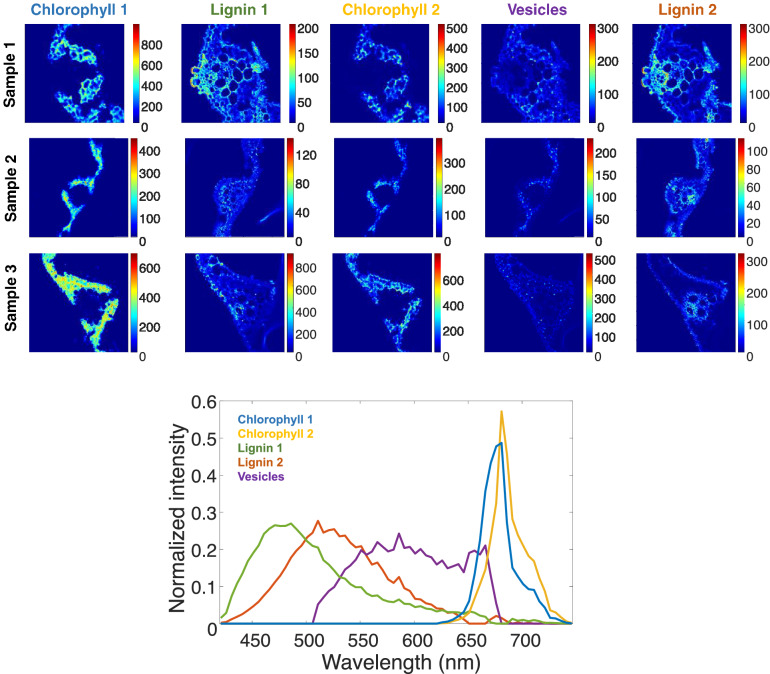
Bottom plot, pure fluorescence spectra profiles. It can be observed five different components. Lignins have an emission in blue and green, while. Vesicles in yellow, and chlorophylls in orange. Top plot, pure distribution maps are showed.

The multiset could be described by five components, as suggested by PCA. In the MCR optimization, the non-negativity constraint was applied to the concentration profiles and spectral signatures of all components because emission spectra are not negative. Considering the prior information mentioned above, selectivity/local rank was also applied to the spectral signature of chlorophylls, set to have null emission in wavelengths lower than 625 nm, and to the component presumably linked to vesicles, set to have null emission below 505 nm and above 680 nm. Figure 5 shows the resolved spectral signatures and the distribution maps of the three samples used in the fusion of images of all platforms.

Five components were identified as natural fluorophores in leaves. Component yellow and blue are identified as chlorophylls, showing a maximum at 682 nm. The maps show clearly that chlorophylls are located at the mesophyll cells, where there are chloroplasts and biochemical activity, such as the photosynthesis^15^. Orange and green components could be lignins, since the emission range observed goes from 430 to 550 nm. As it can be observed in the distribution maps, lignins are present in plant cell walls, plant vascular system and in the epidermis of the leaves^15^. Finally, the purple component, based on its location and its shape as a droplet or vesicle, is presumably identified as a type of body-lipid or body-silica. Yellow fluorescence with a long range can be observed. The characterization of these vesicles was not possible using only the fluorescence emission.

### SR-FTIR HSI multiset analysis

**Figure 6:**
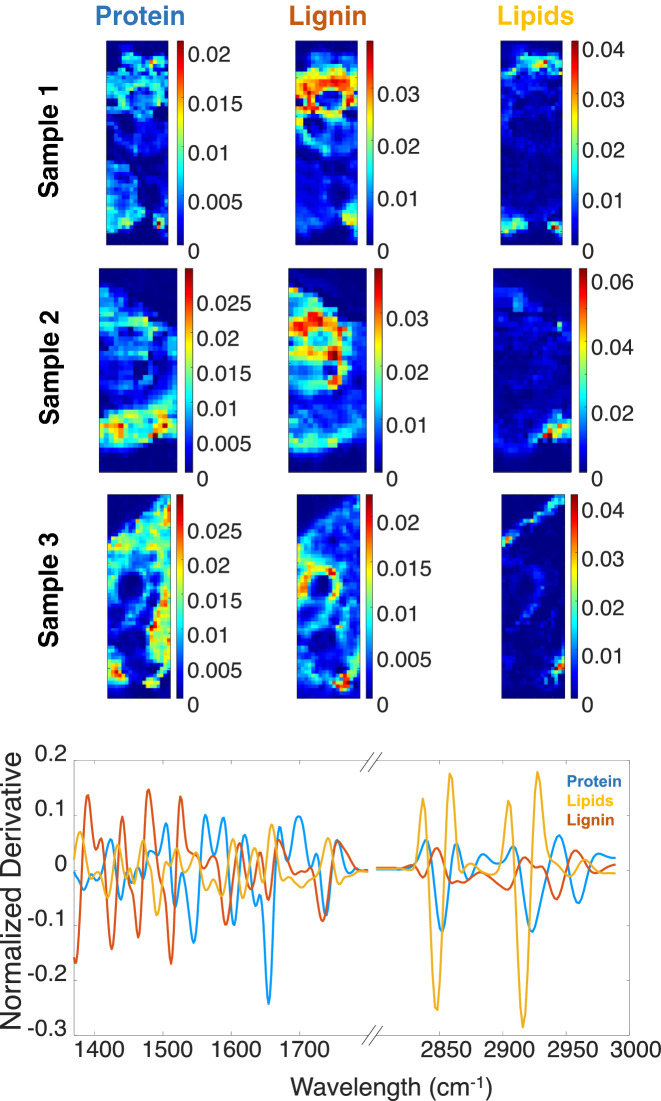
Top plot, pure distribution maps for the three components. Bottom plot, pure SR-FTIR spectral signatures of the component related to lipids (in yellow), to proteins (in blue) and lignin (orange).

Three components were suggested by PCA in all SR-FTIR HSI to describe the multiset. During the iterations, non-negativity was applied only to the concentration profiles of all components because pure signatures have negative values due to the second derivative preprocessing. Figure 6 shows the resolved spectral signatures and the distribution maps of the three samples analyzed. Three components could be identified with distinct IR spectral signatures. The blue component shows mainly protein bands (Amide I (1655 cm^−1^) and Amide II (1543 cm^−1^)^31^. It is possible to observe the location of the proteins mainly in the mesophyll cells, where there are proteins as enzymes related to the biochemical activity. The orange component shows bands associated with lignin (carbonyl (1732 cm^−1^))^32^ and it is possible to observe its presence in the vascular system. The vascular system has cells fortified with lignin to give robustness to the plant. The yellow component is related to lipid bands (methylene groups (2916 and 2846 cm^−1^))^32^. This yellow component is present in the epidermis. Often, leaves show a small layer of resins in their epidermis to avoid water loss. These results are in agreement with the biological compounds naturally present in leaves.

### Raman HSI multiset analysis

**Figure 7:**
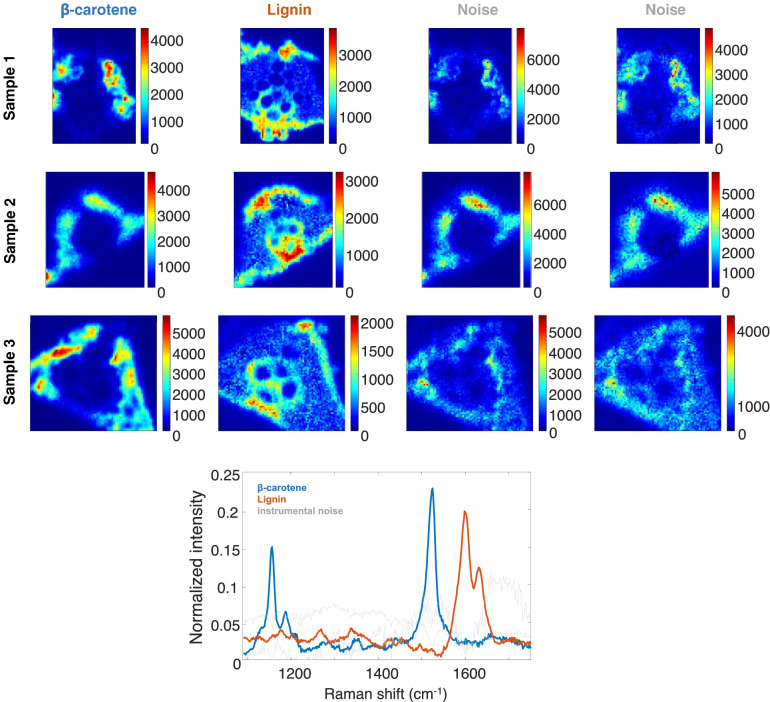
Top plot, pure distribution maps of the resolved components in the three samples. β-carotene, first column and lignin in the second column, the last two columns show the distribution maps related to the noise components. Bottom plot, MCR-ALS resolved pure Raman spectra profiles related to carotenes and lignin. In gray, the two spectral profiles related to noise.

Four components were suggested by PCA in all Raman HSI to describe the multiset. During the iterations, non-negativity constraint was applied to the concentration profiles and the pure spectral signatures of all components. In Fig. 7 it is possible to observe two components associated with biological contributions (in blue and orange) were identified, whereas two additional components (in gray) were attributed to instrumental noise detected in previous works^33^. The blue component was characterized as β-carotene (with typical Raman features at 1155 and 1525 cm^−1^)^34^. β-carotene is a strongly colored red–orange pigment and during photosynthesis β-carotene normally serves as antenna pigments, transferring singlet excitation energy to chlorophyll. Therefore β-carotene can be found at mesophyll cells, where chlorophyll is. The orange characterized component is lignin (with typical Raman features at 1598 and 1631 cm^−1^)^35^. Lignin can be found at the vascular system and sclerenchyma cells, which are strongly lignified.

### Image fusion of fluorescence, SR-FTIR and Raman HSI

MCR-ALS was applied to identify in a complete way the constituents present in the rice leaves. Several MCR-ALS models were tested with different number of components. Six components were needed to explain the relevant variation in images. Adding more components did not provide additional interpretable information. Several initial estimates based on SIMPLISMA or on the connection of resolved signatures coming from multisets of individual techniques were tested.

In a data fusion, constraints can be applied in **C**_*i*_ and **S**_*i*_^T^ submatrices in different ways. In all analyses, non-negativity was applied to concentration profiles. Non-negativity was applied to fluorescence and Raman **S**_*i*_^T^ profiles, whereas SR-FTIR profiles were left unconstrained. Table 2 shows the identification of the six components resolved in the definitive MCR-ALS model. This identification was useful to set local rank constraints.

**Table 2:** Summary of the components identified and the imposed selectivity/local rank.

Component	Identified as	Local rank constraint in ${\mathbf{S}}^{{\text{T}}}$ spectral range (forced to be zero)		
		Fluorescence	SR-IR	Raman
1	Chlorophyll	420–625	–	All*
2	Lignin 1	–	–	–
3	Lipids	505–680	–	All*
4	Protein	All*	–	All*
5	β-carotene	All*	–	–
6	Lignin 2	–	–	–

*No signal was detected for the technique in the related component.

Note that for infrared interval, no selectivity constraint was imposed. Several components were forced to be zero (labelled ‘All’) in certain techniques. Finally, for lipids and chlorophyll, some spectral regions of the fluorescence spectra were forced to be zero.

Several components are not detected by all techniques with the instrumental parameters used in this work. Thus, chlorophyll and lipids do not have Raman signal according to previous measurements. The contribution most linked to proteins in SR-FTIR does not have either fluorescence or Raman signal. Furthermore, fluorescence from β-carotene was not detected. For these reasons, selectivity/local rank was applied to force null signals in all components that are not detected in the suitable technique. Other local rank constraints related to spectral regions with null fluorescence were also applied in the analysis of the multiset of all fused techniques.

**Figure 8:**
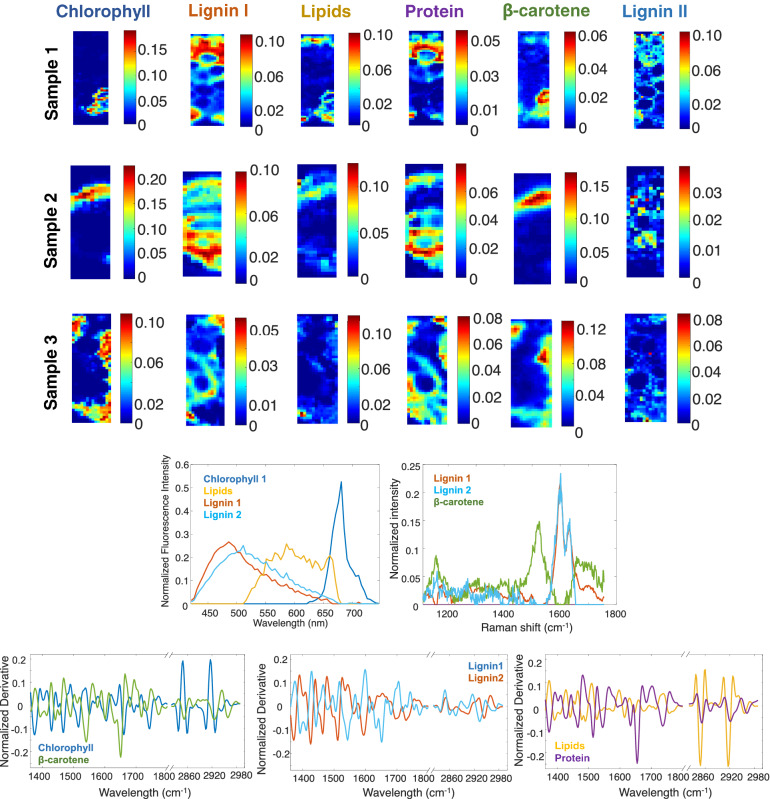
Top, pure distribution maps of the six components identified with the fused images for the three samples. Bottom, pure spectral signatures of fluorescence, Raman and SR-FTIR. SR-FTIR spectra were broken down in plots of two components for better visualization.

The results of the MCR-ALS analysis are shown in Fig. 8. Six components were identified. The blue component was characterized as chlorophyll. It is possible to observe in the distribution maps that chlorophyll is located at mesophyll cells on all samples. The fluorescence pure signature exhibits a typical emission spectrum of chlorophyll with a maximum of 682 nm^15^. The infrared spectrum has bands that could be related to the chlorophyll structure (alkanes (2930 to 2840 cm^−1^), ester (1741 cm^−1^) and alkenes (1660 cm^−1^). The green component was characterized as β-carotene. The component was located at mesophyll cells in distribution maps and Raman pure signature shows the typical Raman peaks at 1525 and 1157 cm^−1^^34^. As is expected, β-carotene appears in the same leaf zone as chlorophyll. This component also shows a relevant protein band in SR-FTIR. This may be the consequence of β-carotenes binding specifically to some protein receptors. Orange and cyan components were characterized as types of lignin. Lignin can be observed on concentration map at vascular tissues. The presence of lignin was high in the sclerenchyma cells as well. For the orange component, the pure fluorescence spectrum has a maximum at 487 nm, while a maximum at 512 nm is observed for the cyan lignin. The pure infrared spectrum of the orange component has a band with maximum at 1730 cm^−1^ (carbonyl), but it was not observed for the cyan component. The pure Raman signatures of both components have two typical Raman features from lignin (1632 cm^−1^ and 1601 cm^−1^)^35^. The yellow component was identified as rich in lipids. The pure fluorescence spectral signature coincides with the pure signature presumably attributed to the epidermis and the vesicles. The SR-FTIR pure signature confirms the identity of this component, with two strong peaks at the lipid region (2916 and 2848 cm^−1^)^32^. Vesicles were not possible to be clearly observed in the concentration maps, probably due to the spatial binning.

The purple component was identified as rich in proteins. The pure infrared spectrum shows a strong peak at 1655 cm^−1^, typically of the group amide of the proteins^31^. This component was located in structural regions in distribution map. This result can indicate that these proteins could interact with plant structural elements.

As it can be observed, the fusion gives richer information than the individual analysis since the distinction of components becomes easier due to their now extended multitechnique spectroscopic signature.

There is a clear synergic effect linked to the compensation of weak properties of a technique by stronger points in another one. For instance, fluorescence images have a good spatial resolution but poor spectroscopic features to identify the nature of many compounds, i.e. lipids were not possible to be identified using only fluorescence images since the fluorescence shape is not selective of functional groups. Instead, the fusion with SR-FTIR images allows characterizing unequivocally this component through characteristic bands in the infrared region, with much richer in spectral features. Along this line, the infrared spectra associated with Raman or fluorescence signatures help to identify molecular compounds (lipids and proteins) linked to typical constituents found in plant tissues (carotenes, lignin, chlorophylls, …).

The image fusion also allows distinguishing components with very similar signatures in a technique taking advantage of the clear distinction of the same components in another fused technique. The lignin components depict this situation. On the one hand, the difference in spectral signatures of the two lignin contributions in fluorescence helps in the distinction of the variants of the same compound in Raman spectroscopy, impossible to achieve when Raman images were analysed alone. On the other hand, the very characteristic Raman features for lignin help to confirm the identity of these components, a task more difficult to do only based on the fluorescence information. The increase in discriminating power is also seen in the six resolved infrared signatures in image fusion, which were reduced to three components when this technique was analyzed alone.

## Conclusions

The operating procedure related to image fusion in a multiplatform scenario has been clearly described and the steps detailed, from the data preprocessing and image matching to the unmixing with MCR-ALS multiset analysis and interpretation of information can be generally applied to perform a complete characterization of the components in any imaged sample. The great benefits of joining different kinds of spectroscopic information for a better morphological and chemical characterization of components has been clearly proven in a case study linked to a vegetal tissue.

The fusion strategy presented is the basic pipeline for many image fusion situations that can be encountered in practice. However, it is relevant to know that fusion approaches are being developed recently to compensate for drawbacks, such as the possible presence of relevant components located in image areas not common to all images or the loss of spatial resolution of some techniques to achieve pixel congruence with techniques that provide a lower level of spatial detail. Additionally, the adaption of algorithms that can combine images providing a linear spectrum per pixel, e.g., Raman, infrared, with others yielding a 2D spectroscopic landscape per pixel, e.g., excitation-emission spectra, has also been proposed. Although these approaches are not generally used yet, they open a new direction to make image fusion even more powerful.

## Supplementary Information

Supplementary Information.

## References

[1] Salzer R, Siesler HW (2014). Infrared and Raman Spectroscopic Imaging.

[2] Amigo, J. M. Hyperspectral and multispectral imaging: Setting the scene. In *Data Handling in Science and Technology*, Vol. 32 (ed. Amigo, J. M.) 3–16 (Elsevier, 2020).

[3] de Juan, A., Gowen, A., Duponchel, L. & Ruckebusch, C. Image fusion. In *Data Handling in Science and Technology*, Vol. 31 (ed. Cocchi, M.) 311–344 (Elsevier, 2019).

[4] Borsoi RA, Imbiriba T, Bermudez JCM (2019). Deep generative endmember modeling: An application to unsupervised spectral unmixing. IEEE Trans. Comput. Imaging.

[5] Palsson B, Sigurdsson J, Sveinsson JR, Ulfarsson MO (2018). Hyperspectral unmixing using a neural network autoencoder. IEEE Access.

[6] Bioucas-Dias, J. M. & Plaza, A. An overview on hyperspectral unmixing: Geometrical, statistical, and sparse regression based approaches. In *2011 IEEE International Geoscience and Remote Sensing Symposium*, 1135–1138 (2011).

[7] de Juan A, Tauler R (2021). Multivariate curve resolution: 50 years addressing the mixture analysis problem - a review. Anal. Chim. Acta.

[8] de Juan A, Maeder M, Tauler R, Brown S, Tauler R, Walczak R (2020). Multiset data analysis: Extended multivariate curve resolution. Comprehensive Chemometrics.

[9] de Juan, A. Multivariate curve resolution for hyperspectral image analysis. In *Data Handling in Science and Technology*, Vol. 32 (ed. Amigo, J. M.) 115–150 (Elsevier, 2020)

[10] Dobigeon, N., Altmann, Y., Brun, N. & Moussaoui, S. Linear and nonlinear unmixing in hyperspectral imaging. In *Data Handling in Science and Technology*, Vol. 30 (ed. Ruckebusch, C.) 185–224 (Elsevier, 2016).

[11] Piqueras S (2018). Handling different spatial resolutions in image fusion by multivariate curve resolution-alternating least squares for incomplete image multisets. Anal. Chem..

[12] Bedia C, Sierra À, Tauler R (2020). Application of chemometric methods to the analysis of multimodal chemical images of biological tissues. Anal. Bioanal. Chem..

[13] Gómez-Sánchez A, Marro M, Marsal M, Loza-Alvarez P, de Juan A (2020). 3D and 4D image fusion: Coping with differences in spectroscopic modes among hyperspectral images. Anal. Chem..

[14] Mas S (2019). Use of physiological information based on grayscale images to improve mass spectrometry imaging data analysis from biological tissues. Anal. Chim. Acta.

[15] Donaldson L (2020). Autofluorescence in plants. Molecules.

[16] Gierlinger N, Keplinger T, Harrington M (2012). Imaging of plant cell walls by confocal Raman microscopy. Nat. Protoc..

[17] Movasaghi Z, Rehman S, Rehman DI (2008). Fourier transform infrared (FTIR) spectroscopy of biological tissues. Appl. Spectrosc. Rev..

[18] Yu P (2003). Chemical imaging of microstructures of plant tissues within cellular dimension using synchrotron infrared microspectroscopy. J. Agric. Food Chem..

[19] Savitzky A, Golay MJ (1964). Smoothing and differentiation of data by simplified least squares procedures. Anal. Chem..

[20] Eilers PH (2004). Parametric time warping. Anal. Chem..

[21] Jolliffe, I. T. Principal components in regression analysis. In *Principal Component Analysis* 129–155 (Springer, 1986).

[22] Windig W, Guilment J (1991). Interactive self-modeling mixture analysis. Anal. Chem..

[23] Bro R, De Jong S (1997). A fast non-negativity-constrained least squares algorithm. J. Chemom. J. Chemom. Soc..

[24] Tauler R, Smilde A, Kowalski B (1995). Selectivity, local rank, three-way data analysis and ambiguity in multivariate curve resolution. J. Chemom..

[25] de Juan A, Maeder M, Hancewicz T, Tauler R (2008). Use of local rank-based spatial information for resolution of spectroscopic images. J. Chemom. J. Chemom. Soc..

[26] de Juan A, Maeder M, Hancewicz T, Tauler R (2005). Local rank analysis for exploratory spectroscopic image analysis. Fixed size image window-evolving factor analysis. Chemom. Intell. Lab. Syst..

[27] Hugelier S, Devos O, Ruckebusch C (2015). On the implementation of spatial constraints in multivariate curve resolution alternating least squares for hyperspectral image analysis. J. Chemom..

[28] Ghaffari M, Hugelier S, Duponchel L, Abdollahi H, Ruckebusch C (2019). Effect of image processing constraints on the extent of rotational ambiguity in MCR-ALS of hyperspectral images. Anal. Chim. Acta.

[29] Piqueras S, Maeder M, Tauler R, de Juan A (2017). A new matching image preprocessing for image data fusion. Chemom. Intell. Lab. Syst..

[30] Jaumot J, de Juan A, Tauler R (2015). MCR-ALS GUI 2.0: New features and applications. Chemom. Intell. Lab. Syst..

[31] Krimm S, Bandekar J (1986). Vibrational spectroscopy and conformation of peptides, polypeptides, and proteins. Adv. Protein Chem..

[32] Heredia-Guerrero JA (2014). Infrared and Raman spectroscopic features of plant cuticles: a review. Front. Plant Sci..

[33] Olmos V (2018). Combining hyperspectral imaging and chemometrics to assess and interpret the effects of environmental stressors on zebrafish eye images at tissue level. J. Biophotonics.

[34] Tschirner N (2009). Resonance Raman spectra of β-carotene in solution and in photosystems revisited: an experimental and theoretical study. Phys. Chem. Chem. Phys..

[35] Zhang X, Chen S, Xu F (2017). Combining Raman imaging and multivariate analysis to visualize lignin, cellulose, and hemicellulose in the plant cell wall. J. Vis. Exp. JoVE.

