# Supplementary material for: Linear unmixing protocol for hyperspectral image fusion analysis applied to a case study of vegetal tissues
Source: Sci Rep. 2021 Sep 20;11:18665. doi: 10.1038/s41598-021-98000-0 (PMC8452694; doi:10.1038/s41598-021-98000-0)
Supplement: Supplementary file 1 — Supplementary Information. [file 41598_2021_98000_MOESM1_ESM.pdf]

Supplementary material

LINEAR UNMIXING PROTOCOL FOR HYPERSPECTRAL IMAGE FUSION ANALYSIS APPLIED TO A CASE STUDY OF VEGETAL TISSUES

Adrián Gómez-Sánchez[1],*, Mónica Marro[2], Maria Marsal[2], Sara Zacchetti[1,3], Rodrigo Rocha de Oliveira[1], Pablo Loza-Alvarez[2], Anna de Juan[1]*

Abstract

The supporting information includes S1 illustrate image related to the preprocessing of each spectroscopic technique.

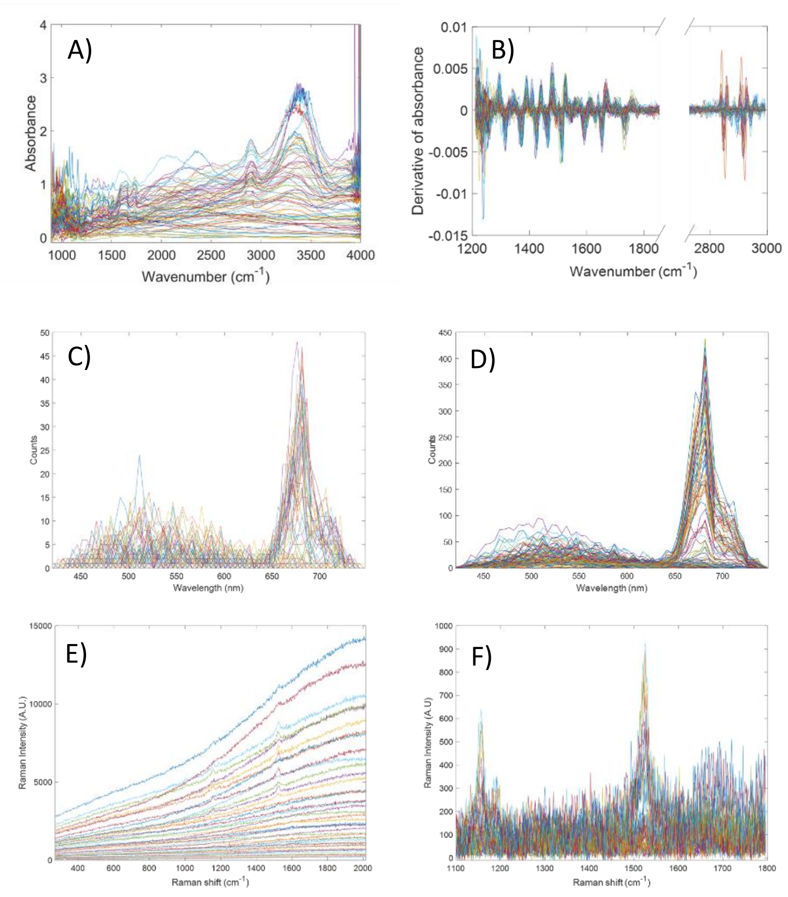

Figure S1. Image preprocessing. A) Raw synchrotron infrared spectra. B) Spectra after preprocessing. C) Original fluorescence spectra. D) Fluorescence spectra after binning. E) Raw Raman spectra. F) Preprocessed Raman spectra.
